# Proteolysis regulates cardiomyocyte maturation and tissue integration

**DOI:** 10.1038/ncomms14495

**Published:** 2017-02-17

**Authors:** Ryuichi Fukuda, Felix Gunawan, Arica Beisaw, Vanesa Jimenez-Amilburu, Hans-Martin Maischein, Sawa Kostin, Koichi Kawakami, Didier Y. R. Stainier

**Affiliations:** 1 Department of Developmental Genetics, Max Planck Institute for Heart and Lung Research, Ludwigstrasse 43, 61231 Bad Nauheim, Germany; 2 Division of Molecular and Developmental Biology, National Institute of Genetics, Yata 1111, Mishima, Shizuoka 411-8540, Japan

## Abstract

Tissue integrity is critical for organ formation and function. During heart development, cardiomyocytes differentiate and integrate to form a coherent tissue that contracts synchronously. However, the molecular mechanisms regulating cardiac tissue integrity are poorly understood. Here we show that proteolysis, via the E3 ubiquitin ligase ASB2, regulates cardiomyocyte maturation and tissue integrity. Cardiomyocytes in *asb2b* zebrafish mutants fail to terminally differentiate, resulting in reduced cardiac contractility and output. Mosaic analyses reveal a cell-autonomous requirement for Asb2b in cardiomyocytes for their integration as *asb2b* mutant cardiomyocytes are unable to meld into wild-type myocardial tissue. *In vitro* and *in vivo* data indicate that ASB2 negatively regulates TCF3, a bHLH transcription factor. TCF3 must be degraded for cardiomyocyte maturation, as TCF3 gain-of-function causes a number of phenotypes associated with cardiomyocyte dedifferentiation. Overall, our results show that proteolysis has an important role in cardiomyocyte maturation and the formation of a coherent myocardial tissue.

## Abstract (web summary)

Proper heart development and synchronous cardiomyocyte contraction rely on tissue integrity. Here the authors show that the ubiquitin-proteasome system and the E3 ubiquitin ligase ASB2 in particular are crucial for cardiomyocyte maturation and tissue integrity through the degradation of the TCF3 transcription factor.

The development of functional organs requires tissue integrity, which is achieved through the coordinated regulation and positioning of cell–cell contacts^1^,^2^. Cardiac tissue integrity is particularly important for cardiac function as the myocardial wall must withstand stress from mechanical forces^3^. Understanding the molecular mechanisms underlying tissue integrity is also crucial to develop effective tissue engineering protocols to generate functional and coherent myocardial tissue^4^. During cardiomyocyte differentiation, progenitor cells initiate the expression of sarcomeric genes^5^,^6^,^7^, and as cardiomyocytes mature they continue to develop more organized sarcomeric structures^8^, as well as highly specialized cell–cell junctions termed intercalated disks^9^. Mature sarcomeres and cell–cell junctions are essential for mechanical and electrical coupling of cardiomyocytes, and allow for synchronized contractions^9^. Although cardiomyocyte specification and differentiation are well-studied^5^,^6^, the molecular mechanisms regulating the maturation of sarcomeres and cell–cell junctions to maintain cardiac tissue integrity remain poorly understood. Proteolysis is essential for various cellular phenomena and this process is mainly mediated by the ubiquitin-proteasomal system (UPS)^10^,^11^. Ankyrin repeat and SOCS box containing 2 (ASB2) is an E3 ubiquitin ligase that functions to specify target proteins for proteasome-dependent degradation. ASB2 is expressed in haematopoietic and muscle cells, including the heart^12^; however, its function in the heart is unknown.

Here we show that ASB2 functions to regulate cardiomyocyte maturation and myocardial tissue integrity. Three-dimensional (3D) imaging of hearts using several zebrafish transgenic lines shows that *asb2b* mutant cardiomyocytes exhibit disorganized sarcomeres and cell–cell junctions, as well as defects in apico-basal polarity, resulting in reduced cardiac contractility and output. Moreover, we show that ASB2 induces the proteasome-dependent degradation of TCF3. TCF3 overexpression in cardiomyocytes leads to the disassembly of sarcomeres, disruption of cell–cell junctions and upregulation of immature markers, phenotypes associated with cardiomyocyte dedifferentiation. We propose that proteolysis via ASB2 regulates cardiomyocyte maturation and myocardial tissue integrity through the regulation of TCF3.

## Results

### *asb2b* mutant zebrafish exhibit cardiac defects

**Figure 1:**
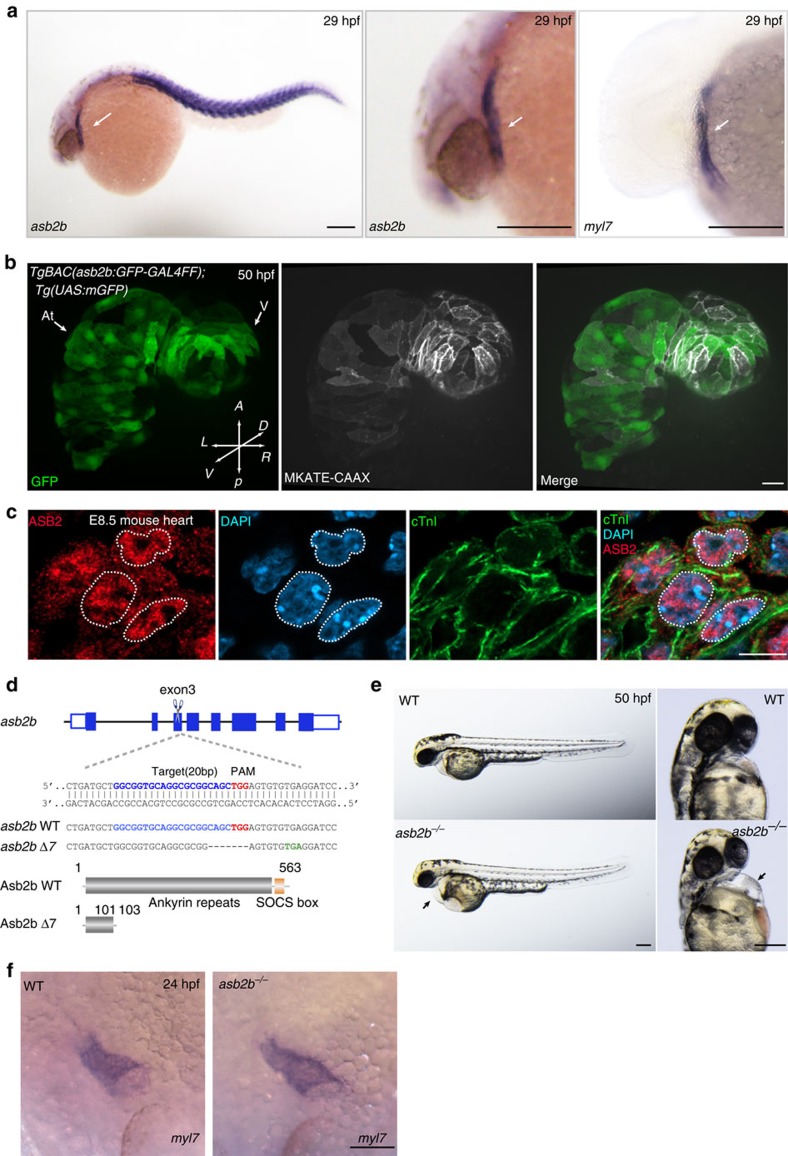
***asb2b*** **mutant zebrafish exhibit cardiac defects.** (**a**) *In situ* hybridization for *asb2b* and *myl7* expression in 29 hpf embryos. *asb2b* is expressed in the *myl7*^+^ heart tube (arrows) and in the somites. Lateral views, anterior to the left. Scale bars, 200 μm. (**b**) Three-dimensional images of 50 hpf *Tg(asb2b:GFP-GAL4FF);Tg(UAS:mGFP);Tg(myl7:MKATE-CAAX)* hearts. Scale bar, 40 μm. (**c**) E8.5 mouse heart co-stained for ASB2 (red), cardiac troponin I (cTnI, green) and 4,6-diamidino-2-phenylindole (DAPI; blue), showing nuclear and cytoplasmic localization of ASB2; dashed lines outline nuclei. Scale bars, 10 μm. (**d**) Schematic representation of the *asb2b* locus. The gRNA target sequence, PAM and premature stop codon are highlighted in blue, red and green, respectively. Deleted nucleotides are indicated by dashes. Predicted structure of WT and mutant (Asb2b Δ7) proteins. The *asb2b Δ7* allele (*asb2b*^*bns33*^) is predicted to encode a truncated polypeptide containing two incorrect amino acids (102–103 aa). (**e**) Bright-field micrographs of 50 hpf WT and *asb2b* mutant embryos in lateral and ventral views. Black arrows point to pericardial edema. Scale bars, 200 μm (**f**) *in situ* hybridization for *myl7* expression in 24 hpf WT and *asb2b* mutant. Scale bar, 100 μm. At, atrium; *A*, anterior; *D*, dorsal; *L*, left; *P*, posterior; *R*, right; V, ventricle; *V*, ventral.

We initially investigated the global effects of proteasome inhibition on cardiomyocyte maturation. We treated 24 h post fertilization (hpf) zebrafish embryos, whose cardiomyocytes already exhibit nascent sarcomeres, with bortezomib, a proteasome inhibitor, for 72 h. We found that the treated animals exhibited mild pericardial oedema and defects in myofilament maturation, without obvious cardiomyocyte death or morphological defects (Supplementary Fig. 1a). To gain insight into the molecular mechanisms that mediate proteasome-dependent proteolysis during cardiomyocyte maturation, we examined the expression of various E3 ubiquitin ligase genes in developing zebrafish hearts. We found that *asb2b*, whose expression starts shortly after cardiac specification (Supplementary Fig. 1b), becomes more highly expressed at later stages in the heart tube and somites (Fig. 1a), whereas its paralogue *asb2a* is expressed in somites and the head region (Supplementary Fig. 1c). To better examine *asb2b* expression, we generated a green fluorescent protein (GFP) bacterial artificial chromosome (BAC) reporter line, *TgBAC(asb2b:GFP-GAL4FF)*, whose expression mimics *asb2b* expression as observed by *in situ* hybridization (Supplementary Fig. 1d). When observed in combination with a cardiomyocyte-specific reporter line, *Tg(myl7:MKATE-CAAX)*^13^, we found GFP expression in the cardiomyocytes (Fig. 1b). We also examined the subcellular localization of ASB2 in mouse embryonic cardiomyocytes by immunostaining and found it in the nucleus and cytoplasm (Fig. 1c). To examine the role of zebrafish *asb2a* and *asb2b*, we generated loss-of-function mutants using the CRISPR/Cas9 technology (Fig. 1d and Supplementary Fig. 1e). We found that *asb2b* mutants (*asb2b*^*bns33*^) exhibit pericardial oedema, whereas *asb2a* mutants (*asb2a*^*bns144*^) appear unaffected (Fig. 1e and Supplementary Fig. 1f). We also examined *asb2a*; *asb2b* double mutants and found no significant differences in the severity and frequency of pericardial oedema compared with *asb2b* single mutants (Supplementary Fig. 1f). Thus, we focused our attention on the function of *asb2b* in cardiomyocytes and found that *asb2b* mutant hearts display reduced cardiac function, as evidenced by defects in fractional shortening and reduced blood flow velocity (Supplementary Fig. 1g). These phenotypes could not be attributed to defects in the cardiac conduction system, as we examined cardiac-specific calcium activation using the *Tg(myl7:gCaMP)* line^14^ and found no obvious changes in calcium excitation between wild type (WT) and *asb2b* mutant hearts (Supplementary Movies 1 and 2). Furthermore, the expression of several cardiac differentiation marker genes appeared to be unaffected in *asb2b* mutants (Fig. 1f, Supplementary Fig. 1h and Supplementary Table 1), suggesting that the cardiac phenotypes observed are not due to defects in the early specification of cardiac cells.

### *asb2b* mutant cardiomyocytes exhibit structural defects

**Figure 2:**
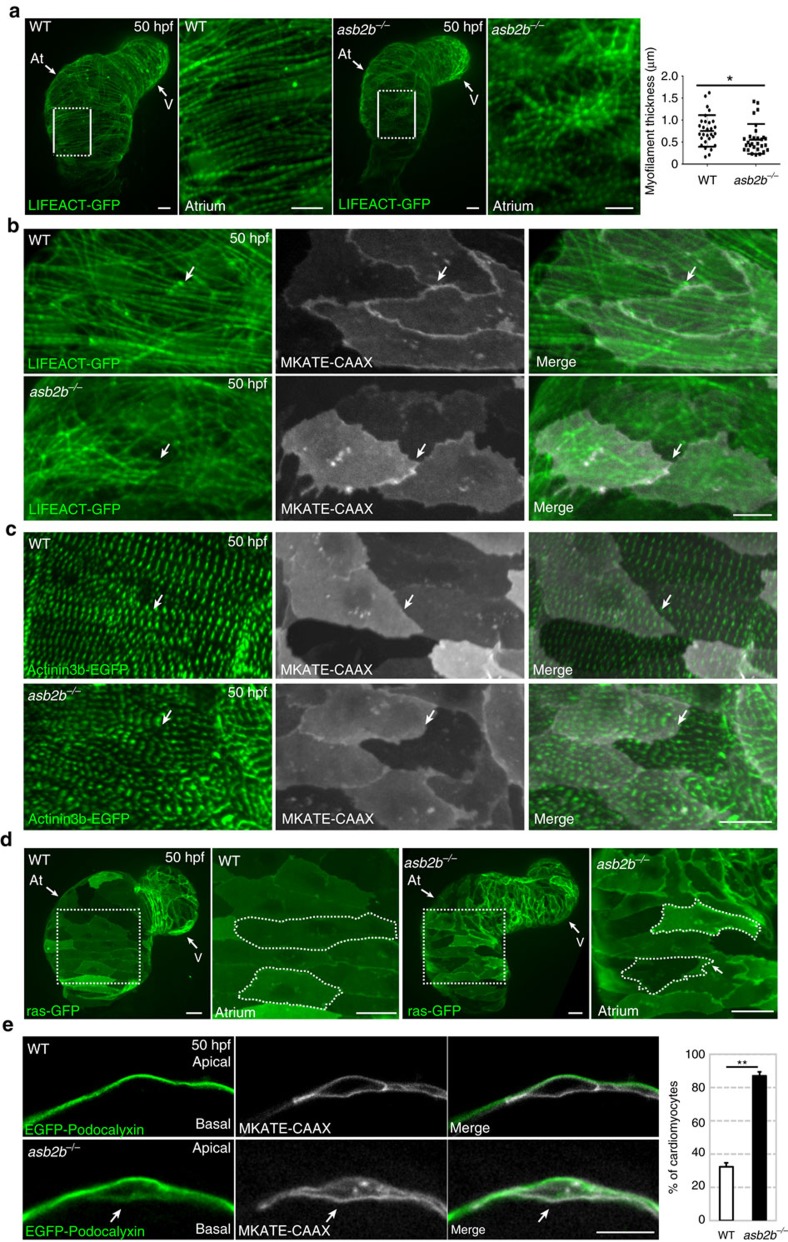
***asb2b*** **mutant cardiomyocytes exhibit structural defects.** (**a**) Three-dimensional images of 50 hpf *Tg(myl7:LIFEACT*-*GFP)* WT and *asb2b* mutant hearts reveal differences in myofilament thickness and organization. Magnified views of myofilaments in white boxes are shown on the right. Myofilament thickness was measured in 50 hpf WT and *asb2b* mutant hearts (*n*=29 myofilaments from 5 hearts). (**b**) Three-dimensional images of 50 hpf *Tg(myl7:LIFEACT-GFP);Tg(myl7:MKATE-CAAX)* WT and *asb2b* mutant atria. The myofilaments in WT are organized across cell borders (arrows), whereas *asb2b* mutant hearts show disorganized myofilaments between adjacent cardiomyocytes (arrows). (**c**) Three-dimensional images of 50 hpf *Tg(myl7:actn3b*-*EGFP)* WT and *asb2b* mutant atria. Atrial cardiomyocytes in WT exhibit an organized *z*-band pattern across cell–cell borders (arrows), whereas those in *asb2b* mutants exhibit a disorganized *z*-band pattern (arrows). (**d**) Three-dimensional images of 50 hpf *Tg(myl7:ras-GFP)* WT and *asb2b* mutant hearts. Magnified views of myofilaments in white dotted boxes are shown on the right. Cardiomyocytes are outlined to define shape. (**e**) Single-plane images of 50 hpf *Tg(myl7:EGFP-Podocalyxin);Tg(myl7:MKATE-CAAX)* WT and *asb2b* mutant atria. In WT, EGFP-Podocalyxin is localized on the abluminal (apical) side of cardiomyocytes, whereas in *asb2b* mutants, it appears to be localized on both the abluminal and luminal (basal) sides (arrows). Number of cardiomyocytes exhibiting EGFP-Podocalyxin localization on the basal side in WT and *asb2b* mutant hearts (*n*=5 hearts, with averages taken from 20 cells per heart). *P<0.05 and **P<0.01 by one-way analysis of variance (ANOVA) followed by Tukey's honest significant difference test. Error bars, s.e.m. Scale bars, 20 μm. At, atrium; V, ventricle.

Cardiac contractility is generated by sarcomeres, the basic contractile units of muscle^8^. To examine sarcomeres in detail, we crossed the *asb2b* mutant with the *Tg(myl7:LIFEACT-GFP*) line^15^ to label myofilaments. Atrial cardiomyocytes in WT hearts contain well-organized myofilaments, which appear mostly aligned with one another. In contrast, myofilaments in *asb2b* mutants are thinner, disorganized and misaligned (Fig. 2a). Accordingly, immunostaining revealed mislocalization of myosin heavy chain in *asb2b* mutant hearts (Supplementary Fig. 2a) and further ultra-structural analysis confirmed the thinner sarcomeres in *asb2b* mutant hearts (Supplementary Fig. 2b). To analyse myofilament organization between neighbouring cardiomyocytes, we used the *Tg(myl7:MKATE-CAAX*) line to label cardiomyocyte cell membranes. We observed that myofilaments in the WT atrium appear to be continuous across neighbouring cardiomyocytes, whereas those in *asb2b* mutants exhibit discontinuous myofilaments, with noticeable breaks observed at cell–cell borders (Fig. 2b). In concordance with these observations, analysis of α-Actinin3b-EGFP expression, which highlights *z*-bands^13^, reveals disorganized alignments between mutant cardiomyocytes (Fig. 2c). On the other hand, we found that forced overexpression of *asb2b* in cardiomyocytes also resulted in disrupted myofilaments (Supplementary Fig. 2c), indicating that tight regulation of *asb2b* expression levels is crucial for cardiac development.

To examine cardiomyocyte morphology in greater detail, we used a cardiomyocyte-specific membrane-targeted GFP reporter line (*Tg(myl7:ras-GFP)*)^16^. We observed that *asb2b* mutant cardiomyocytes exhibit irregular cell–cell borders and membrane protrusions that overlap with neighbouring cardiomyocytes (Fig. 2d and Supplementary Fig. 3a), as well as ectopic Cdh2 (aka N-cadherin)-tdTomato punctae in the protruding membranes (Supplementary Fig. 3b). This phenotype was not accompanied by obvious changes in the expression levels of N-cadherin, a main junction molecule in cardiomyocytes^3^, or in the expression levels of other junctional components (Supplementary Fig. 3c,d and Supplementary Table 1). We also examined myocardial apico-basal polarity using a cardiomyocyte-specific transgenic line expressing Podocalyxin, an apical protein^17^, fused to enhanced GFP (EGFP). EGFP-Podocalyxin localizes specifically to the apical (abluminal) side of WT cardiomyocytes, but becomes mislocalized to the basal (luminal) side in *asb2b* mutant cardiomyocytes (Fig. 2e). We also observed that EGFP-Podocalyxin strongly accumulates in protruding membranes in *asb2b* mutant cardiomyocytes (Supplementary Fig. 3e). As the shape of *asb2b* mutant cardiomyocytes was reminiscent of that of migrating cells, we examined the localization of the pleckstrin homology domain of Akt1, which localizes to the leading edge of migrating cells^18^. We found enriched localization of pleckstrin homology-Akt in membrane protrusions in *asb2b* mutant cardiomyocytes (Supplementary Fig. 3f). Furthermore, we found upregulation in the expression of several genes involved in actin rearrangement and cell migration in *asb2b* mutant hearts (Supplementary Fig. 3g and Supplementary Table 1)^19^. These data suggest that *asb2b* mutant cardiomyocytes exhibit changes in cell morphology and cell–cell junctions, as well as loss of apico-basal. These cell morphological phenotypes observed in *asb2b* mutant cardiomyocytes probably contribute to the functional defects described earlier.

### *asb2b* functions cell autonomously in cardiomyocytes

**Figure 3:**
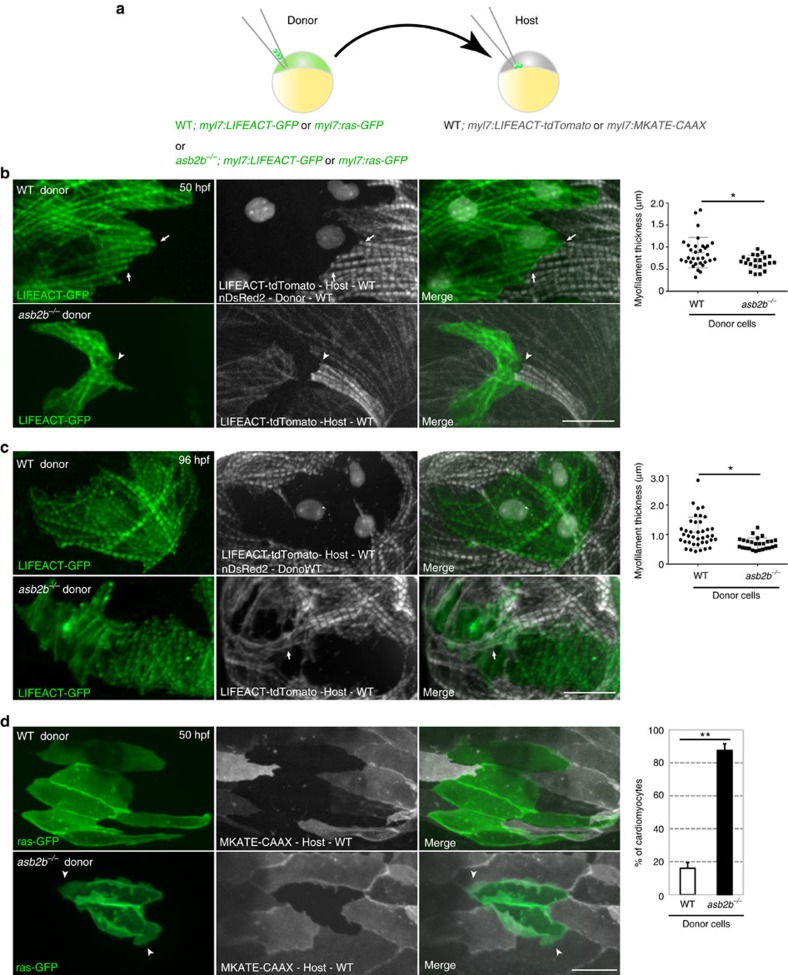
***asb2b*** **functions cell autonomously in cardiomyocytes.** (**a**) Schematic representation of the transplantation experiments. (**b**) Three-dimensional images of 50 hpf chimeric atria. *Tg(myl7:LIFEACT-GFP);Tg(myl7: nDsRed2)* WT or *Tg(myl7:LIFEACT-GFP*) *asb2b* mutant donor cells were transplanted into *Tg(myl7:LIFEACT-tdTomato*) WT host embryos. WT donor cardiomyocytes show integration of the myofilaments into WT hearts (arrows), whereas those from *asb2b* mutants fail to meld into WT hearts (arrowheads). Myofilament thickness was measured in 50 hpf WT and *asb2b* mutant donor cardiomyocytes (*n=*32 (WT) and 22 (mutant) myofilament from 5 hearts). (**c**) Three-dimensional images of 96 hpf chimeric atria. *asb2b* mutant cardiomyocytes in WT hearts fail to integrate their myofilaments with those of adjacent cardiomyocytes. WT host cardiomyocytes appear to establish their myofilaments underneath *asb2b* mutant cardiomyocytes (arrows). Myofilament thickness was measured in 96 hpf WT and *asb2b* mutant donor-derived cardiomyocytes (*n*=39 (WT) and 26 (mutant) myofilament from 5 hearts). (**d**) Three-dimensional images of 50 hpf chimeric atria resulting from transplanting WT or *asb2b* mutant cells from *Tg(myl7:ras-GFP*) donors into *Tg(myl7:MKATE-CAAX*) WT hosts. *asb2b* mutant cardiomyocytes exhibit abnormal membrane protrusions (arrowheads). Number of WT and *asb2b* mutant donor-derived cardiomyocytes exhibiting membrane protrusions in 50 hpf WT hearts (*n*=5 hearts, with averages taken from 30 cardiomyocytes per heart.). *P<0.05 and **P<0.01 by one-way analysis of variance (ANOVA) followed by Tukey's honest significant difference test. Error bars, s.e.m. Scale bars, 20 μm.

Contractility and blood flow have both been reported to regulate cardiomyocyte shape during development^20^,^21^. To rule out the possibility that defects in *asb2b* mutant hearts arise due to secondary effects from decreased contractility and blood flow, we carried out cell transplantations to examine the behaviour of *asb2b* mutant cardiomyocytes in WT hearts (Fig. 3a), whose contractility and resulting blood flow velocity were not affected by the presence of mutant cells (Supplementary Fig. 4a and Supplementary Movies 3 and 4). We found that *asb2b* mutant cardiomyocytes in WT hearts failed to develop organized myofilaments and also exhibited thin myofilaments that appeared discontinuous with the myofilaments of adjacent WT host cardiomyocytes (Fig. 3b and Supplementary Fig. 4b). At later stages, WT host cardiomyocytes assembled myofilaments and connected exclusively with each other, while excluding *asb2b* mutant cardiomyocytes (Fig. 3c). Altogether, these data suggest a cell-autonomous function for Asb2b in cardiomyocyte maturation and integration. We also observed that *asb2b* mutant cardiomyocytes in WT hearts exhibited irregular borders and membrane protrusions (Fig. 3d). Thus, these results indicate that the phenotype observed in *asb2b* mutant hearts is due to a failure in cardiomyocyte integration and not secondary to cardiac dysfunction.

### TCF3 is a target of ASB2 in cardiomyocytes

**Figure 4:**
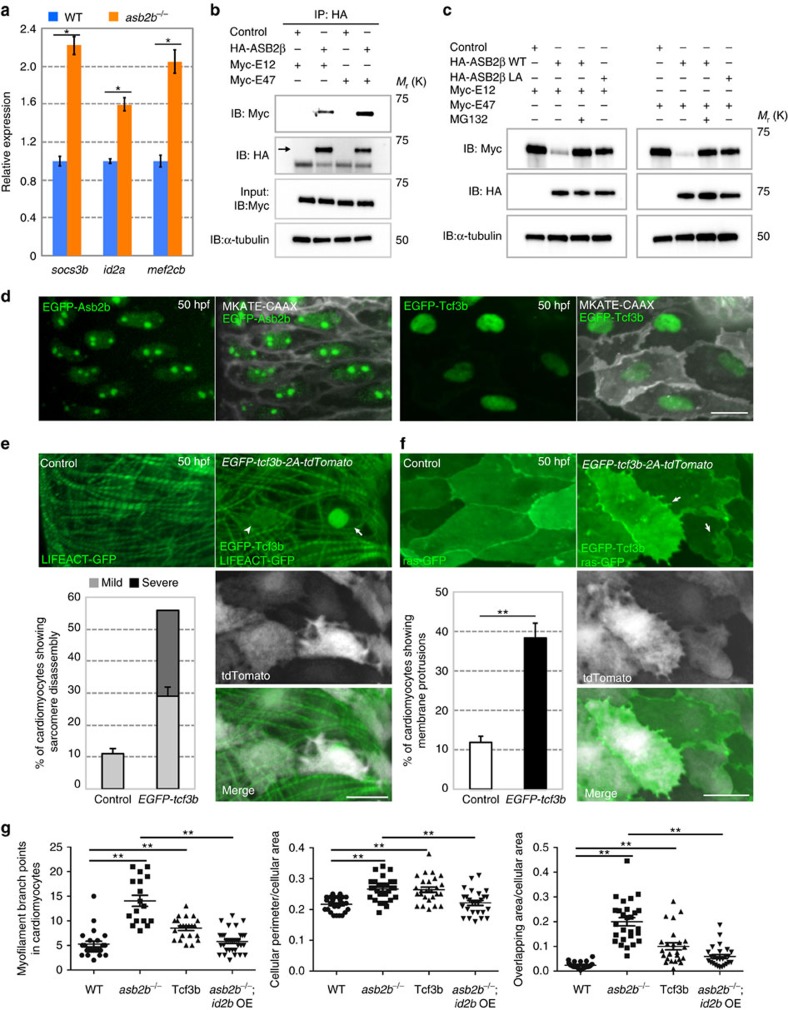
TCF3 is a target of ASB2 in cardiomyocytes. (**a**) Relative mRNA expression (qPCR) of *socs3b*, *id2a* and *mef2cb* in isolated 50 hpf WT (blue bar) and *asb2b* mutant (orange bar) hearts (*n*=2 technical replicates; RNA samples obtained from 500 isolated hearts). (**b**) Co-immunoprecipitation (IP) of HA-ASB2β and Myc-E12 or Myc-E47 immunoblotted (IB) for HA (arrow) or Myc. Rat NCMs were transfected with *HA-Asb2β* or control (mock), together with *Myc-E12* or *Myc-E47* adenovirus vectors. All adenovirus vectors used contain an independent GFP cassette to assess transfection efficiency. After 24 h of transfection, cells were treated with 20 μM MG132 for 8 h and then IP was performed; lower bands in HA blot are HA antibody heavy chains. (**c**) Asb2β induced E12 and E47 degradation in a proteasome-dependent manner. Rat NCMs were transfected with control, *HA-Asb2β* or *HA-Asb2β L595A* mutant (*Asb2βLA*), together with *Myc-E12* or *Myc-E47* adenovirus vectors. After 48 h of transfection, NCMs were treated with 20 uM MG132 or DMSO for 12 h and these protein samples were extracted for immunoblotting. (**d**) Three-dimensional images of a 50 hpf *TgBAC(asb2b:GFP-asb2b);Tg(myl7:MKATE-CAAX)* atrium, and a *Tg(myl7:MKATE-CAAX)* atrium expressing EGFP-Tcf3b. Both Asb2b and Tcf3 localize to the nucleus in cardiomyocytes. (**e**) Three-dimensional images of 50 hpf *Tg(myl7:LIFEACT-GFP*) and *Tg(myl7:LIFEACT-GFP);Tg(myl7:EGFP-tcf3b-2A-tdTomato)* atria. (The transgenic fish exhibit mosaic expression of EGFP-tcf3b-2A-tdTomato in the heart.) EGFP-Tcf3b-2A-tdTomato expressing cardiomyocytes exhibit disassembly of myofilaments. Arrow points to a severe case of myofilament disassembly, arrowhead to a mild case. Number of cardiomyocytes exhibiting sarcomere disassembly (*n*=5 hearts, with averages taken from 20 cardiomyocytes per heart). (**f**) Three-dimensional images of 50 hpf *Tg(myl7:ras-GFP*) or *Tg(myl7:ras-GFP);Tg(myl7:GFP-tcf3b-2A-tdTomato)* atria. Tcf3b-2A-tdTomato expressing cardiomyocytes exhibit abnormal membrane protrusions (arrows). Number of cardiomyocytes exhibiting membrane protrusions (*n*=5 hearts, with averages taken from 20 cardiomyocytes per heart). (**g**) Quantification of myofilament branch points, cellular morphology and membrane protrusions in 50 hpf WT, *asb2b* mutant, *Tg(myl7:GFP-tcf3b-2A-tdTomato)* WT and *Tg(myl7:id2b-2A-tdTomato) asb2b* mutant atrial cardiomyocytes in *Tg(myl7:LIFEACT-GFP)* or *Tg(myl7:ras-GFP)* background. (*n*=15 to 34 cardiomyocytes from 3 to 6 hearts). *P<0.05 and **P<0.01 by one-way analysis of variance (ANOVA) followed by Tukey's honest significant difference test. Error bars, s.e.m. Scale bars, 20 μm.

ASB2 is a subunit of the E3 ubiquitin ligase complex^22^ that functions to recognize specific substrates and target them for degradation by the proteasome. Thus, we hypothesized that the loss of *asb2b* function results in an increase of particular substrates, which causes the phenotype observed in the cardiomyocytes. We first examined the global effect of the loss of *asb2b* on transcriptional pathways by microarray analysis using isolated hearts from WT and *asb2b* mutants. Using Ingenuity Pathway Analysis ( www.ingenuity.com), we found that TCF3 (also termed E2A), which was previously reported as a substrate of the haematopoietic-specific isoform ASB2α in mammals^22^, was a predicted upstream regulator of differentially expressed genes. Accordingly, downstream targets of Tcf3 were upregulated in *asb2b* mutant hearts (Fig. 4a and Supplementary Table 1). In mammalian muscle, the ASB2β isoform is predominantly expressed^12^, but there have been no reports that ASB2β targets TCF3. Thus, we performed immunoprecipitation and found that both E12 and E47, major splice isoforms of TCF3 (ref. 23), can interact with ASB2β in rat neonatal cardiomyocytes (NCMs, Fig. 4b). We also found that ASB2β induces the proteasome-dependent degradation of both E12 and E47, which could be inhibited by the proteasome inhibitor MG132 (Fig. 4c). Furthermore, the point mutant ASB2βLA, which is defective in forming an E3 ubiquitin ligase complex^24^, failed to induce TCF3 degradation (Fig. 4c). It was previously reported that ASB2α cellular localization was dependent on the localization of its substrate^24^. We examined the cellular localization of EGFP-Asb2b and EGFP-Tcf3b, and found that both localized to the nucleus in zebrafish cardiomyocytes (Fig. 4d). We also observed that EGFP-Asb2b strongly accumulated in 4,6-diamidino-2-phenylindole-negative subnuclear structures (Supplementary Fig. 4c). Next, to test the hypothesis that the *asb2b* mutant phenotype was caused by an increase in Tcf3 levels, we generated a stable transgenic line overexpressing *EGFP-tcf3b-2A-tdTomato* in cardiomyocytes. Cardiomyocytes overexpressing Tcf3b exhibited severe myofilament disassembly or disorganized myofilaments, similar to the phenotype observed in *asb2b* mutant cardiomyocytes (Fig. 4e), as well as irregular cell shape and membrane protrusions (Fig. 4f). To quantitatively assess changes in the degree of myofilament organization and cellular morphology in Tcf3b-overexpressing cardiomyocytes, we counted the number of myofilament branch points, which represent the points where the direction of myofilaments changes, and also measured cellular area and perimeter (Fig. 4g). We observed a significant difference between WT and Tcf3b-overexpressing cardiomyocytes, suggesting that Tcf3b overexpression in *asb2b* mutant hearts causes the phenotype described above. Furthermore, we hypothesized that inhibition of Tcf3 function *in vivo* could rescue the cardiomyocyte phenotypes in *asb2b* mutants. The Id family of transcription factors is known to act as dominant-negative HLH proteins for TCF3 (ref. 23). To inhibit Tcf3 function, we generated a transgenic line overexpressing *Id2b-2A-tdTomato* in cardiomyocytes and crossed this line to the *asb2b* mutant. We found that Id2b overexpression led to a reduction in the severity of pericardial oedema (Supplementary Fig. 5a) as well as a recovery of cell morphology and myofilament organization (Fig. 4g and Supplementary Fig. 5b), indicating that suppression of Tcf3 results in partial rescue of the *asb2b* mutant phenotype. Altogether, these data suggest that an important function of Asb2b is to regulate the levels of Tcf3, and that higher than WT levels of Tcf3 during cardiomyocyte maturation causes defects in cardiomyocyte integrity.

### TCF3 causes dedifferentiation of rat NCMs

**Figure 5:**
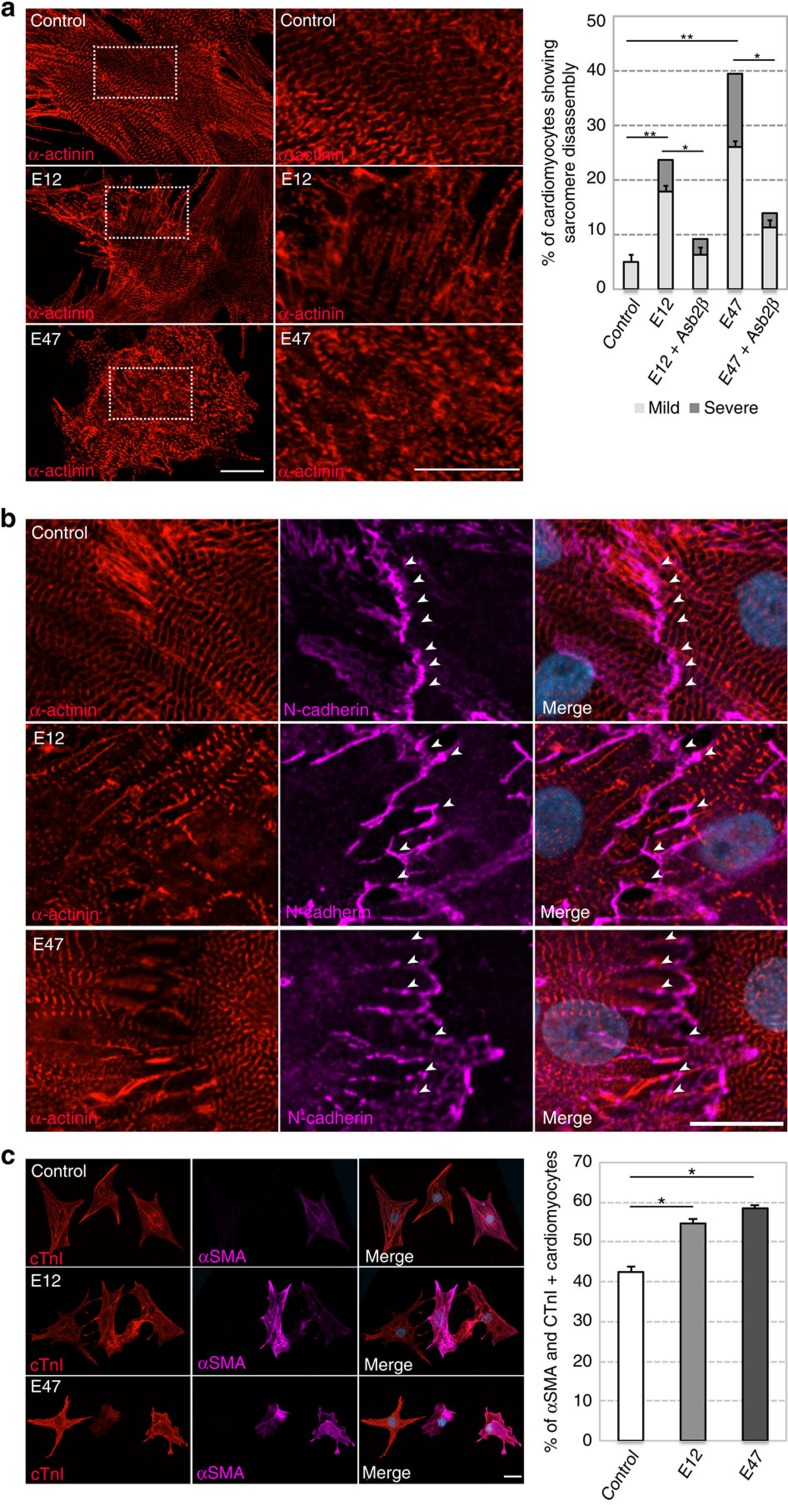
TCF3 causes dedifferentiation of rat NCMs. (**a**) Rat NCMs transfected with control, *E12* or *E47* adenovirus vectors, stained for sarcomeric-α-actinin (red). Magnified views in white dotted boxes are shown on the right. Number of cardiomyocytes transfected with adenovirus vectors exhibiting sarcomere disassembly (*n*=2, with averages taken from 50 cardiomyocytes). (**b**) Rat NCMs transfected with control, *E12* or *E47* adenovirus vector, co-stained for α-actinin (red), N-cadherin (magenta) and 4,6-diamidino-2-phenylindole (DAPI; blue). Cardiomyocytes expressing E12 and E47 exhibit disruptions of cell–cell junction (arrowheads). (**c**) Rat NCMs transfected with control, *E12* or *E47* adenovirus vector, co-stained for cardiac troponin I (cTnI, red), α-smooth muscle actin (α-SMA, magenta) and DAPI (blue). Number of cardiomyocytes transfected with adenovirus vectors exhibiting both cTnI and α-SMA expression (*n*=2, with averages taken from 50 cells). *P<0.05 and **P<0.01 by one-way analysis of variance (ANOVA) followed by Tukey's honest significant difference test. Error bars, s.e.m. Scale bars, 20 μm.

Next, we wanted to determine whether TCF3 inhibits cardiomyocyte maturation and/or acutely induces disruptions in cardiomyocytes that have already established sarcomeres and cell–cell junctions. To this end, we cultured rat NCMs until they exhibited synchronous contraction, indicative of coordinated sarcomeres and cell–cell junctions. Overexpression of both E12 and E47 by adenoviral transfection resulted in the disassembly of sarcomeres (Fig. 5a) and this effect was rescued by co-transfection with ASB2β (Fig. 5a). We also observed a disruption of cell–cell junctions (Fig. 5b), as observed in zebrafish *asb2b* mutants. Moreover, we found that TCF3 protein levels in mouse hearts decreased during development (Supplementary Fig. 5c), further suggesting that TCF3 must be negatively regulated to allow cardiomyocyte maturation. Interestingly, we found the upregulation of markers associated with stem cells and cardiac progenitors, including *gata4* (ref. 5), *tbx5b*^5^, *kita* (an orthologue of mammalian *c-kit*^25^) and *vim* (*vimentin*^26^) in *asb2b* mutant hearts compared with WT (Supplementary Fig. 5d). Furthermore, we examined the expression of immature markers in rat NCMs overexpressing TCF3 and found that a typical fetal marker, α*a*-smooth muscle actin^27^ (Fig. 5c), as well as Vimentin (Supplementary Fig. 5e), were upregulated. These phenotypes, observed both *in vitro* and *in vivo*, are reminiscent of cardiomyocyte dedifferentiation^25^,^26^,^27^,^28^.

## Discussion

In this study, we identified an important role for ASB2 in cardiomyocytes for the maturation and organization of their sarcomeres and cell–cell junctions, processes that are essential for myocardial tissue integrity. These findings reveal key molecular mechanisms regulating cardiomyocyte maturation. In chimeric hearts, *asb2b* mutant cardiomyocyte failed to meld with WT myocardium, suggesting that *asb2b* functions cell-autonomously to ensure proper cell morphological characteristics that affect overall myocardial tissue integrity. Cell recognition and cell–cell adhesion are mediated in part by cadherin molecules, and cells expressing the same type and similar levels of cadherin molecules preferentially adhere to one another^29^. However, *asb2b* mutant cardiomyocytes do not exhibit obvious changes in N-cadherin expression, suggesting that other mechanisms of cell recognition and adhesion are at play.

We identified TCF3 as a main target of ASB2 responsible for the defects in *asb2b* mutant cardiomyocytes. Our results show that overexpression of TCF3 recapitulates the *asb2b* mutant phenotypes. These phenotypes, namely disassembly of sarcomeres, disruption of cell–cell junctions and re-expression of progenitor markers, have previously been reported in dedifferentiating cardiomyocytes in mouse neonatal and adult hearts, as well as during newt and zebrafish heart regeneration^25^,^26^,^27^,^30^. We also observed that the expression levels of cardiac differentiation marker genes were not affected in *asb2b* mutants, suggesting that the mutant cardiomyocytes may be in an intermediate, partially differentiated state. Taken together, our data suggest that in *asb2b* mutants, high levels of TCF3 block cardiomyocyte maturation. TCF3 may also cause an epithelial to mesenchymal transition-like process in cardiomyocytes, as TCF3 can induce epithelial to mesenchymal transition in epithelial cells^23^.

During cardiomyocyte specification, cardiac progenitors migrate extensively to form the primitive heart tube^6^,^7^. After this initial migration, motility has to be suppressed to allow the maturation of cardiomyocytes and their full integration into a coherent tissue, to develop a fully formed and functional organ^6^,^7^,^8^. MESP1 plays a key role in the specification of the cardiac lineage^31^ and it was recently reported that it has to dimerize with TCF3 to regulate downstream target genes^32^. Our results indicate that TCF3 must subsequently be suppressed to allow cardiomyocyte maturation, and that this suppression is, in part, mediated by its proteolysis via the UPS. Regulation of TCF3 function via proteolysis may allow cardiomyocytes to quickly transition from one state to the next.

On the other hand, mechanical forces are known to be involved in cardiomyocyte development and maturation^20^,^21^,^33^. ASB2 is a member of the ankyrin repeat and SOCS box-containing protein family. It has been previously reported that the ankyrin repeats exhibit elastic properties and spring-like behaviour^34^,^35^ and recently the ankyrin repeats of NOMPC (No mechanoreceptor potential C), a transient receptor potential family protein, were shown to be essential for its role in mechanotransduction^36^. Moreover, ASB2 expression increases during mouse embryonic development and myogenic differentiation in C2C12 myoblasts^12^. Therefore, it is possible that the function of ASB2 is linked to mechanical forces during heart development and maturation.

In summary, our data reveal the importance of the UPS and its regulation of a specific transcription factor, for cardiomyocyte maturation and myocardial tissue integrity, and we speculate that proteolysis regulates other developmental transitions that need to occur quickly and efficiently.

## Methods

### Zebrafish

Zebrafish were grown and maintained under standard conditions, as approved by institutional (Max Planck Society) and national ethical and animal welfare guidelines. *Tg(myl7:MKATE-CAAX)*^*Sd11*^ (ref. 13), *Tg(myl7:EGFP-Has.HRAS)*^*s883*^ (ref. 37) (abbreviated *Tg(myl7:ras-GFP)*), *Tg(myl7:LIFEACT-GFP)*^*s974*^ (ref. 15), *Tg(-5.1myl7:DsRed2-NLS*)^f2Tg^ (ref. 16) (abbreviated *Tg(myl7:nDsRed2)*) , *Tg(UAS:mGFP)*^*m1230*^ (ref. 38), *Tg(myl7:gCaMP)*^*s878*^ (ref. 14), *Tg(myl7:actn3b-EGFP)*^*sd10Tg*^ (ref. 13) and *Tg(−0.2myl7*: *EGFP-Podocalyxin*)^*bns103*^ (abbreviated *Tg(myl7:EGFP-Podocalyxin)*) lines were used. *Tg(myl7:LIFEACT-tdTomato)*^*bns141*^ was generated using the same strategy as *Tg(myl7:LIFEACT-GFP)*^*s974*^ (ref. 15). To generate *Tg(myl7:N-cadherin-tdtomato)*^*bns78*^, *Tg(myl7:EGFP-tcf3b-2A-tdTomato)*^*bns142*^ and *Tg(myl7:id2b-2A-tdTomato)*^*bns143*^, complementary DNA fragments of *N-cadherin* (NCBI accession number: NM_131081), *tcf3b* (NCBI accession number: XM_005170492) and *id2b* (NCBI accession number: NM_199541 ) were isolated by RT–PCR, using the following primers: *N-cadherin* forward 5′-GAATTCACCATGTACCCCTCCGGAGGCGTG-3′ and *N-cadherin* reverse 5′-ACGCGTGTCGTCGTTACCTCCGTAC-3′; *tcf3b* forward 5′-GACGAGCTGTACAAGATGAACGAGCCACACCAG-3′ and *tcf3b* reverse 5′-GTCGACCATGTGACTGACGGAGTTG-3′; *id2b* forward 5′-AAGCTTCCACCATGAAGGCAGTCA-3′ and *id2b* reverse 5′GTCGACACGAGACAGGGCTATGAG-3′. *EGFP-tcf3b* or *id2b* PCR fragments and *2A-tdtomato* fragments were combined into one fragment via PCR and cloned into a vector containing Tol2 elements and two I-SceI restriction enzyme sites under the control of the *myl7* promoter element. The *Tg(asb2b:GFP-GAL4FF)*^*bns107*^ reporter line, encoding *GFP* fused *GAL4FF*^39^ and *Tg(asb2b:GFP-asb2b)*^*bns106*^, which carry GFP inserted in-frame with the coding region of *asb2b*, were generated using the BAC clone (CH1073-416D2) using standard recombineering technology^40^. For whole-mount immunostaining, antibodies against myosin heavy chain 1:500 (MF20, eBioscience) and N-cadherin 1:250 (ab12221, Abcam) were used. After washing with PBS, samples were stained with fluorescent secondary antibodies (Life Technologies). For quantifying cardiac function in embryonic zebrafish, movies of beating hearts were taken using a Zeiss Spinning disc CSU-X1 confocal microscope with a high-speed camera, which were then used to calculate fractional shortening as described previously^41^. For the analysis of myocardial-specific calcium activation, one-cell stage *Tg(myl7:gCaMP)*^14^ WT and *asb2b* mutant embryos were injected with *tnnt2a* MO^42^ to stop cardiac contraction for data acquisition and imaged at 50 hpf using a Zeiss spinning disk confocal microscope with CSU-X1 (Yokogawa) and ORCA-flash4.0 sCMOS camera (Hamamatsu). For electron microscopy (EM) analysis, zebrafish embryos were fixed with 3% glutaraldehyde (Merck), followed by 4% OsO4 in 0.1 mM sodium cacodylate buffer (pH 7.4). After dehydration in ethanol and propylene oxide, they were embedded in Epon as described. Ultrathin sections were stained with uranyl acetate and lead citrate, and viewed and photographically recorded under a JEM 1400 electron microscope. To count myofilament branch points, Imaris (Bitplane) was used for 3D image processing and counting. To analyse cellular morphology, ImageJ ( http://rsbweb.nih.gov/ij/) was used to measure cellular area and perimeter.

### Mouse

For immunostaining, E8.5 mouse embryos were fixed with 4% paraformaldehyde. Antibodies against ASB2 1:100 (HPA001546, Sigma) and anti-cTnI 1:500 (ab56357, Abcam) were used. For western blotting, E8.5, E14.5, P1 and P7 mouse hearts were isolated. Antibodies against TCF3 1:1,000 (G-2, Santa Cruz Biotechnology) were used.

### Genome editing

pT7-gRNA and pT3TS-nCas9n were used to generate sgRNA and Cas9 messenger RNA, respectively. Genomic RNAs (gRNAs) were designed targeting *asb2a* (Supplementary Fig. 1) and *asb2b* (Fig. 2) using CRISPR Design ( http://crispr.mit.edu).

### Cell culture analysis

Rat neonatal (P1) hearts were removed and placed in PBS; they were then minced into small pieces and placed in Hank's balanced salt solution (Gibco) supplemented with 0.0125% trypsin and incubated at 4 °C overnight. The minced hearts were subsequently digested with 1.5 mg ml^−1^ collagenase/dispase mix (Roche) in L-15 medium (Sigma) for 20 min at 37 °C with gentle agitation^43^. Cells were plated onto 0.1% gelatin-coated (Sigma) plates and cultured in DMEM/F12 (Gibco) supplemented with 5% horse serum, L-glutamine, Na-pyruvate, penicillin and streptomycin at 37 °C and 5% CO_2_. For immunofluorescence, cells were fixed using 4% formaldehyde in PBS for 10 min. Samples were permeabilized with 0.3% Triton X-100 in PBS for 10 min and blocked with 5% fetal bovine serum in PBS for 1 h. Samples were incubated with the following antibodies: anti-cTnI 1:500 (ab56357, Abcam), anti-α-actinin 1:500 (EA-53, Sigma), anti-Vimentin 1:500 (c-20, Santa Cruz Biotechnology) and anti-N-cadherin 1:250 (ab12221, Abcam). After washing with PBS, samples were stained with fluorescent secondary antibodies (Life Technologies) followed by 4,6-diamidino-2-phenylindole staining, to visualize nuclei. Adenovirus vectors for transfection were generated using the AdEasy system (Agilent Technologies). The cDNA fragments of *HA-Asb2β* (NCBI accession number: NM_001011984), *myc-E12* (NCBI accession number: NM_133524) and *myc-E47* (NCBI accession number: NM_001035237) were isolated by RT–PCR, using the following primers: *HA-Asb2β* forward 5′-GCGGCCGCACCATGTATCCATATGATGTTCCAGATTATGCTATGTCGACTGAGATCTCCAC-3′ and *HA-Asb2β* reverse 5′-TCTAGATTACTGTGTATTCTCGTATTTCAGG-3′; *myc-E12* and *myc-E47* forward 5′-GCGGCCGCaccatgGAACAAAAGTTGATTTCAGAAGAAGATCTGATGATGAACCAGTCTCAGAGAA-3′ and *myc-E12* and *myc-E47* reverse 5′-tctagaTCACAGGTGCCCAGCTGG-3′. Adenoviruses were applied to the cultured rat NCMs and incubated for 8 h. The transfected cells were then washed and cultured in growth medium. The efficiency of transfection was assessed by GFP expression, encoded within the adenovirus construct.

### Imaging

Zebrafish embryos were mounted in 1% low-melting agarose supplemented with 0.2% (w/v) tricaine to stop heartbeats. The images were acquired using a Zeiss spinning disk confocal microscope system with CSU-X1 (Yokogawa) and ORCA-flash4.0 sCMOS camera (Hamamatsu) or a confocal microscope (LSM800, Zeiss) using an LD C-Apochromat × 40 (1.1 numerical aperture) objective. Imaris (Bitplane) was used for 3D image processing. Myofilament thickness was measured using the ZEN imaging software (Zeiss).

### Protein analysis

Protein extracts were prepared in lysis buffer (150 mM Tris-HCl pH 7.5, 150 mM NaCl, 1% Triton X-100, 0.2% SDS, 1 mM EDTA, 5 mM NaF, 0.1 mM orthovanadate, 1 mM phenylmethylsulfonyl fluoride and 1 μg ml^−1^ of aprotenin). Proteins were separated by SDS–PAGE and transferred to a polyvinylidene difluoride membrane. The following primary antibodies were used: anti-HA 1:1,000 (3F10, Roche), anti-Myc 1:1,000 (9E10, Santa Cruz Biotechnology) and anti-α-tubulin 1:1,000 (T6199, Sigma). For immunoprecipitation, protein extracts were prepared in lysis buffer (150 mM Tris-HCl pH 7.5, 150 mM NaCl, 0.1% NP40, 1 mM EDTA, 5 mM NaF, 0.1 mM orthovanadate, 1 mM phenylmethylsulfonyl fluoride and 1 μg ml^−1^ of aprotinin). Protein extracts were incubated with anti-HA 1:100 (3F10, Roche) and protein G beads (GE). Beads were washed four times with lysis buffer and precipitated proteins were analysed by immunoblotting. For densitometric analysis of western blottings, ImageJ ( http://rsbweb.nih.gov/ij/) was used. All uncropped images related to western blotting data are available in Supplementary Fig. 6.

### RNA analysis

Total RNA extraction was performed using miRNeasy Mini Kit (Qiagen) and purified RNA was used as a template for cDNA synthesis using the SuperScript second strand kit (Life Technologies), according to the manufacturers' instructions. The gene symbols and gene names are listed (Supplementary Table 1). For quantitative real-time PCR, the CFX Connect Real-Time system (Bio-Rad) and DyNAmo colorFlash SYBR green qPCR kit (ThermoFisher Scientific) were used. mRNA expression levels were normalized to *ef1α* (Supplementary Fig. 1f) or *ef1β* (Fig. 4a and Supplementary Fig. 5c). Gene expression levels were relative to those of 17.5 hpf WT embryos (Supplementary Fig. 1f) or 50 hpf WT hearts (Fig. 4a and Supplementary Fig. 5c). The Ct values are shown in Supplementary Table 2. The following primers were used: *myl7* forward 5′-GGAGAGAAGCTCAATGGCACA-3′and *myl7* reverse 5′-GTCATTAGCAGCCTCTTGAACTCA-3′; *vmhc* forward 5′-CTCCTGGTGCAATGGAGAAT-3′ and *vmhc* reverse 5′-CAGGTATGGCTGCAGGATTT-3′; *myh6* forward 5′-TGGCGATGCTGACGTTTCTT-3′ and *myh6* reverse 5′-TCACGGTGACACAAAACAGCC-3′; *asb2b* forward 5′-GACCCAGTGGGACTGAAGTG-3′ and *asb2b* reverse 5′-TGTAGAGTGCGCTTGTCCAC-3′; *socs3* forward 5′-GGGACAGTGAGTTCCTCCAA-3′ and *socs3* reverse 5′-ATGGGAGCATCGTACTCCTG-3′; *id2a* forward 5′-GACTCCAATTCGGCGATAAC-3′ and *id2a* reverse 5′-GTGTCCTGCTGTCCTCTGTG-3′; *mef2cb* forward 5′-CTATGGAAACCACCGCAACT-3′ and mef2cb reverse 5′-CCATTCCCTGTCCTGGTAGA-3′; *gata4* forward 5′-TTACCTGTGCAATGCCTGTG 3′ and *gata4* reverse 5′-TGCAGACTGGCTCTCCTTCT-3′; *tbx5b* forward 5′-GCTCCACCATCTCCTGACTG-3′ and *tbx5b* reverse 5′-TGGTAGCTGAAGAGGGGGTA-3′; *kita* forward 5′-GCCCATGCAACAGAGAAAGA-3′ and *kita* reverse 5′-CGCAGGAAGTTCAACAGGTC-3′; *vim* forward 5′-TCCATGAAGGAAACCAGACC-3′ and *vim* reverse 5′-CTCCAGCGTGGATCTTCAGT-3′; *ef1α* forward 5′-CTTCTCAGGCTGACTGTGC-3′ and *ef1α* reverse 5′-CCGCTAGCATTACCCTCC-3′; *ef1β* forward 5′-ATCTGTTTGGCTCCGATGAG-3′ and *ef1β* reverse 5′-CAGGCTTCTTTGCCTTCTTG-3′. For microarray analysis, total RNA samples were sent to Oak-Labs (Germany), where fluorescent complementary RNA was generated using the Low Input QuickAmp Labeling kit (Agilent Technologies) and subsequently hybridized on a microarray. Fluorescence signals on microarrays were detected by the SureScan Microarray Scanner (Agilent Technologies). The detailed protocol is available from Oak-Labs ( http://www.oak-labs.com/). For whole-mount *in situ* hybridization, digoxygenin-labelled anti-sense RNA probes for *myl7*, *asb2a* and *asb2b* were generated using SP6 or T7 polymerase (Roche) and a DIG RNA Labeling Kit (Roche). cDNA encoding *myl7* was a kind gift from Atsuo Kawahara (University of Yamanashi). To synthesize mRNA or RNA probes for *in situ*, cDNA encoding *asb2a* (NCBI accession number: XM_691298) and *asb2b* (NCBI accession number: NM_214725) were isolated by RT–PCR with the following primers: *asb2b* forward 5′-GAATTCATGACCCGGTTCTCTTATG-3′ and *asb2b* reverse 5′-CTAGATCACAGGTGGTGATGTTCC-3′; *asb2a* forward 5′-GGATCCatggcagcagccagagtgac-3′; *asb2a* reverse 5′-GAATTCctacatttcatattctcgttc-3′

The PCR fragments were subcloned into the pCS2 vector.

### Statistical analyses

Statistical analyses were performed by Student's *t*-test or one-way analysis of variance with Tukey's test. Data were considered statistically significant if the *P*-value was <0.05 (*).

### Data availability

The data that support the findings in this study are available within the article and its Supplementary Information files, and from the corresponding authors upon request.

## Additional information

**How to cite this article:** Fukuda, R. *et al*. Proteolysis regulates cardiomyocyte maturation and tissue integration. *Nat. Commun.* **8,** 14495 doi: 10.1038/ncomms14495 (2017).

**Publisher's note**: Springer Nature remains neutral with regard to jurisdictional claims in published maps and institutional affiliations.

## Supplementary Material

Supplementary Information Supplementary Figures and Supplementary Tables.

Supplementary Movie 1 Calcium imaging of Tg(myl7:gCaMP) WT heart. One-cell stage Tg(myl7:gCaMP) WT embryos were injected with tnnt2a MO and imaged at 50 hpf.

Supplementary Movie 2 Calcium imaging of Tg(myl7:gCaMP) asb2b mutant heart shows no obvious changes in cardiac conduction. One-cell stage Tg(myl7:gCaMP) asb2b mutant embryos were injected with tnnt2a MO and imaged at 50 hpf.

Supplementary Movie 3 Time-lapse imaging of a WT heart transplanted with WT cells. Tg(myl7:LIFEACT-GFP) WT donor cells (green) were transplanted to the margin of Tg(myl7:LIFEACT-tdTomato) WT host embryos (red) at the blastula stage and imaged at 50 hpf.

Supplementary Movie 4 Time-lapse imaging of a WT heart transplanted with asb2b mutant cells. Tg(myl7:LIFEACT-GFP) asb2b mutant donor cells (green) were transplanted to the margin of Tg(myl7:LIFEACT-tdTomato) WT host embryos (red) at the blastula stage and imaged at 50 hpf.
