# Supplementary material for: Proteolysis regulates cardiomyocyte maturation and tissue integration
Source: Nat Commun. 2017 Feb 17;8:14495. doi: 10.1038/ncomms14495 (PMC5321751; doi:10.1038/ncomms14495)
Supplement: Supplementary Information — Supplementary Figures and Supplementary Tables. [file ncomms14495-s1.pdf]

Supplementary Figures

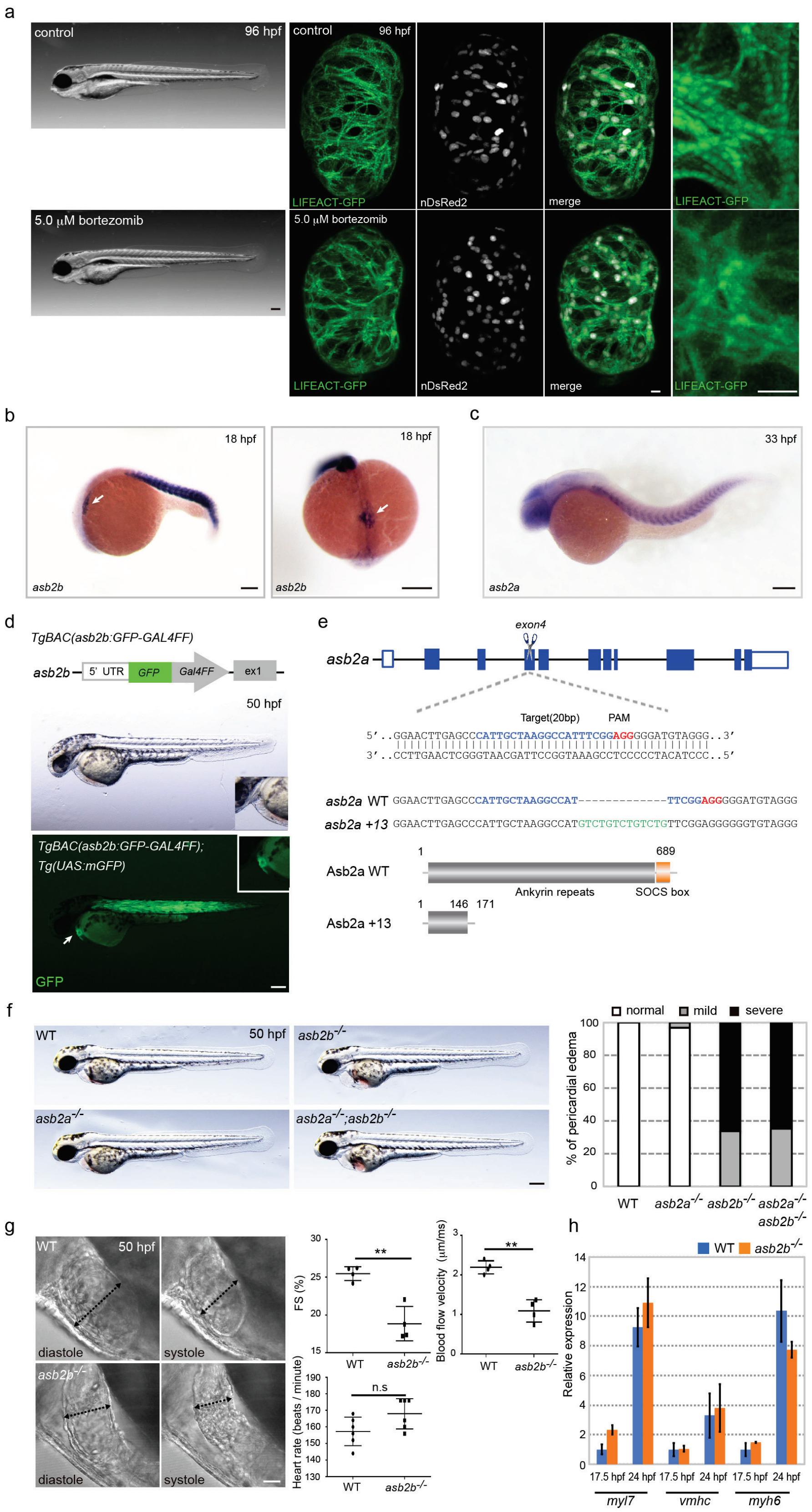

Supplementary Figure 1 - *asb2b* mutant zebrafish exhibit cardiac defects. (a) Bright-field micrographs of 96 hpf larvae treated with DMSO (control) and bortezomib. Lateral

views, anterior to the left. Scale bar, 200 μm. 3D images of 96 hpf *Tg(myl7:LIFEACT-GFP*) hearts in animals treated with DMSO (control) and bortezomib. Animals were treated from 24 until 96 hpf. Scale bar, 20 μm. (b) *in situ* hybridization for *asb2b* expression in 18 hpf embryos. Lateral view, anterior to the left; dorsal view, anterior to the bottom. *asb2b* is expressed in the heart cone (arrows) as well as in the somites. (c) *in situ* hybridization for *asb2a* expression in 33 hpf embryos. Lateral view, anterior to the left. Scale bars, 200 μm. (d) Schematic representation of the reporter transgene: a *GFP-GAL4FF-polyA-Km^r* cassette was inserted at the ATG start codon in an *asb2b* BAC. Lateral views of a 50 hpf *Tg(asb2b:GFP-GAL4FF);Tg(UAS:mGFP)* embryo and close-up views of the heart; anterior to the left. An arrow points to the heart. GFP is expressed in the heart and somites. Scale bar, 200 μm. (e) Schematic representation of *asb2a* locus and the gRNA. Target sequence of gRNA and PAM are highlighted in blue and red, respectively. Inserted nucleotides are indicated in green. Predicted structure of WT and Asb2a +13 mutant proteins. *asb2a +13* allele is predicted to encode a truncated polypeptide containing 25 incorrect amino acids (147-171 aa). (f) Bright-field micrographs of 50 hpf WT, *asb2a* mutant, *asb2b* mutant, and *asb2a*;*asb2b* double mutant embryos in lateral views. Scale bar, 200 μm. percentage of WT and mutant embryos exhibiting pericardial edema (*n*=50 fish). (g) Lateral close-up views of 50 hpf WT and *asb2b* mutant hearts in diastole and systole. Anterior to the left. Two-headed arrows indicate width of the ventricle. Quantification of ventricular fractional shortening (FS) and aortic blood flow velocity in WT and *asb2b* mutants. Heart rate measurement in 50 hpf WT and *asb2b* mutants (*n*=4 to 6 fish). Scale bar, 20 μm. (h) Relative mRNA expression (qPCR) of *myl7*, *vmhc* and *myh6* expression at 17.5 and 24 hpf in WT and *asb2b* mutants (*n*=2 technical replicates, RNA samples were obtained from 50 embryos). $^{**}P<0.01$ by one-way analysis of variance (ANOVA) followed by Tukey's HSD test. Error bars, SEM.

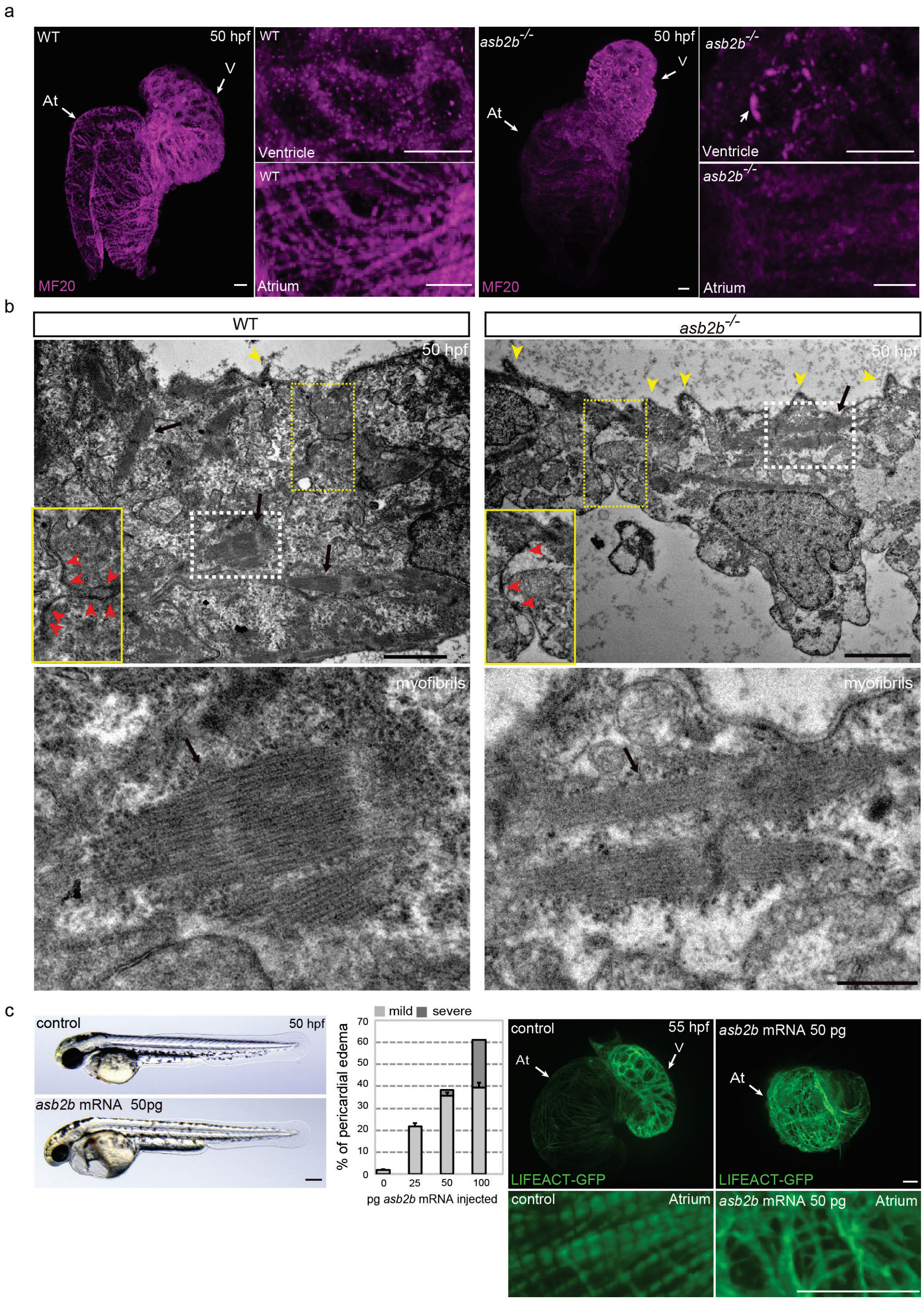

Supplementary Figure 2 - *asb2b* is involved in myofilament organization.
(a) 3D images of 50 hpf WT and *asb2b* mutant hearts stained with myosin heavy chain antibody (MF20). At, atrium; V, ventricle. Cardiomyocytes in the WT ventricle exhibit cortical myosin localization, while those in *asb2b* mutants show less cortical localization of myosin and exhibit punctae of myosin (arrow). WT atrial cardiomyocytes exhibit clear myofilaments, whereas fewer myofilaments are observed in *asb2b* mutants. (b) Electron

micrographs of 50 hpf WT and *asb2b* mutant ventricles. Close-up views of the cardiomyocyte cell-cell contact regions in yellow dotted boxes are shown (yellow squares with red arrowheads). Ventricular cardiomyocytes in *asb2b* mutants exhibit fewer regions of cell-cell contact (red arrowheads). *asb2b* mutant cardiomyocytes exhibit many more membrane protrusions (yellow arrowheads) compared to WT. Scale bars, 2 μm. Magnified views of myofilaments in white dotted boxes are shown (black arrows). Scale bar, 0.5 μm. (c) Lateral views of a 50 hpf control embryo and an embryo injected with 50 pg *asb2b* mRNA. Anterior to the left. Scale bar, 200 μm. Number of fish showing pericardial edema (n=60 fish). 1-cell stage embryos were injected with different amounts of *asb2b* mRNA and analyzed at 55 hpf. 3D images and close-up views of *Tg(myl7:LIFEACT-GFP)* hearts of 55 hpf embryos injected with 50 pg *asb2b* mRNA. At, atrium; V, ventricle. Error bars, SEM. Scale bars, 20 μm.

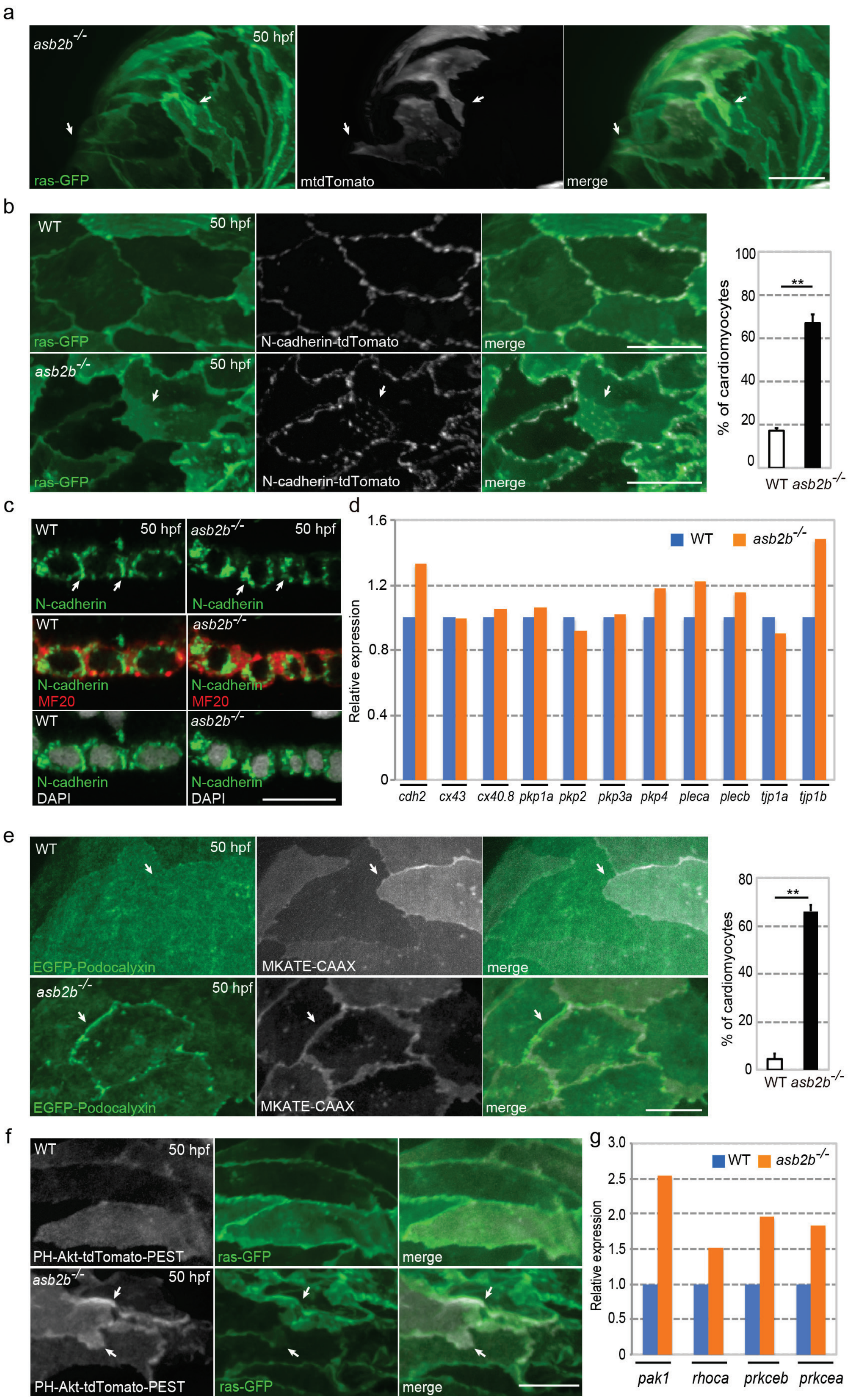

Supplementary Figure 3 – *asb2b* mutant cardiomyocytes exhibit irregular cell-cell junctions. (a) 3D images of 50 hpf *Tg(myl7:ras-GFP) asb2b* mutant atria mosaically expressing membrane-tdTomato (mtdTomato). Mosaic expression of mtdTomato was achieved by injecting a *myl7:mtdTomato* plasmid into *Tg(myl7:ras-GFP) asb2b* mutant

one-cell stage embryos. Membrane protrusions of mtdTomato positive cardiomyocytes are shown (arrows). (b) 3D images of 50 hpf *Tg(myl7:ras-GFP);Tg(myl7:N-cadherin-tdTomato)* WT and *asb2b* mutant atria. N-cadherin-tdTomato molecules in WT cardiomyocytes exhibit lateral localization in cell-cell junctions, while those in *asb2b* mutants also exhibit localization in overlapping regions (arrows). Number of cardiomyocytes exhibiting N-cadherin-tdTomato punctae in overlapping regions with neighbouring cardiomyocytes in 50 hpf WT and *asb2b* mutant hearts (n =5 hearts, with averages taken from 25 cardiomyocytes per heart). (c) Co-staining for N-cadherin (green), Myosin heavy chain (MF20, red) and DAPI (white) in 50 hpf WT and *asb2b* mutant hearts, transverse views. No obvious differences in N-cadherin expression are observed (arrows). (d) Relative mRNA expression (microarray) of junction component genes in isolated 50 hpf WT and *asb2b* mutant hearts. Gene names can be found in Supplementary Table 1. (e) 3D images of 50 hpf *Tg(myl7:EGFP-Podocalyxin);Tg(myl7:MKATE-CAAX)* WT and *asb2b* mutant atria. In WT hearts, EGFP-Podocalyxin shows uniform distribution across cell-cell borders (arrows), while those in *asb2b* mutants exhibit peripheral localization (arrows). Number of cardiomyocytes exhibiting EGFP-Podocalyxin localization in cardiomyocyte cell-cell borders in 50 hpf WT and *asb2b* mutant hearts (n=5 hearts, with averages taken from 20 cardiomyocytes per heart). (f) 3D images of 50 hpf *Tg(myl7:ras-GFP)* WT and *asb2b* mutant atria expressing PH-Akt-tdTomato-PEST. *asb2b* mutant cardiomyocytes exhibit enriched localization of PH-Akt-tdTomato-PEST in membrane protrusions (arrows). (g) Relative mRNA expression (microarray) of *pak1*, *rhoca*, *prkceb* and *prkcea* in isolated 50 hpf WT and *asb2b* mutant hearts. Gene names can be found in Supplementary Table 1. $^{**}P<0.01$ by one-way analysis of variance (ANOVA) followed by Tukey's HSD test. Error bars, SEM. Scale bars, 20 μm.

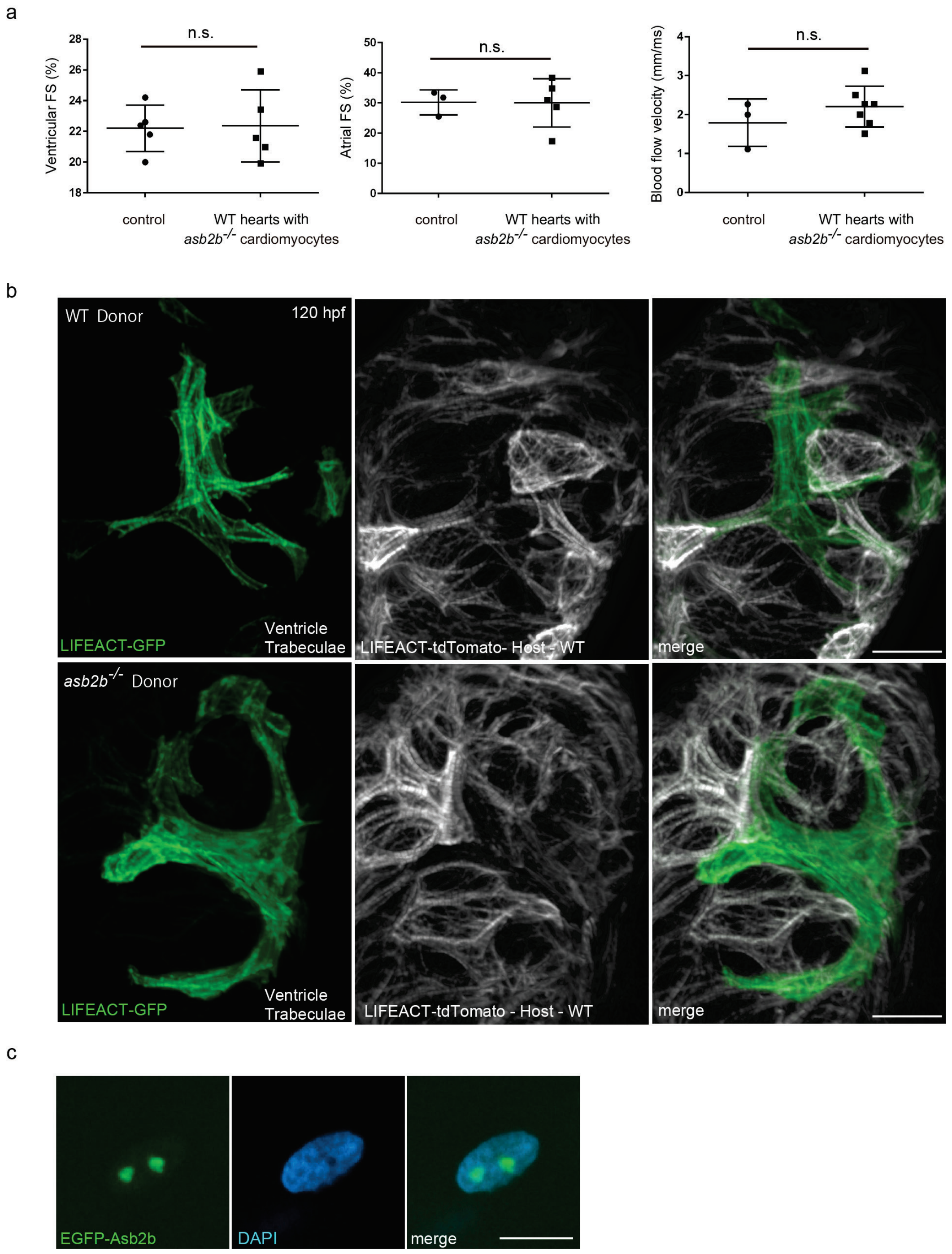

Supplementary Figure 4 – *asb2b* mutant cardiomyocytes in WT hearts exhibit disorganized myofilaments. (a) Quantification of fractional shortening (FS) in ventricles and atria in 50 hpf chimeric hearts, and aortic blood flow velocity. WT or *asb2b* mutant cells were transplanted into WT host embryos. No significant differences in FS or blood flow velocity were observed (*n*=3 to 7 fish). (b) 3D images of 120 hpf chimeric ventricles. *Tg(myl7:LIFEACT-GFP)* WT or *asb2b* mutant donor cells were transplanted into *Tg(myl7:LIFEACT-tdTomato*) WT host embryos. WT donor-derived cardiomyocytes exhibit mature sarcomeres in trabecular cardiomyocytes, while *asb2b* mutant-derived cardiomyocytes exhibit disorganized sarcomeres. (c) A 50 hpf *TgBAC(asb2b:GFP-asb2b)* heart stained with DAPI (blue). Scale bars, 20 μm.

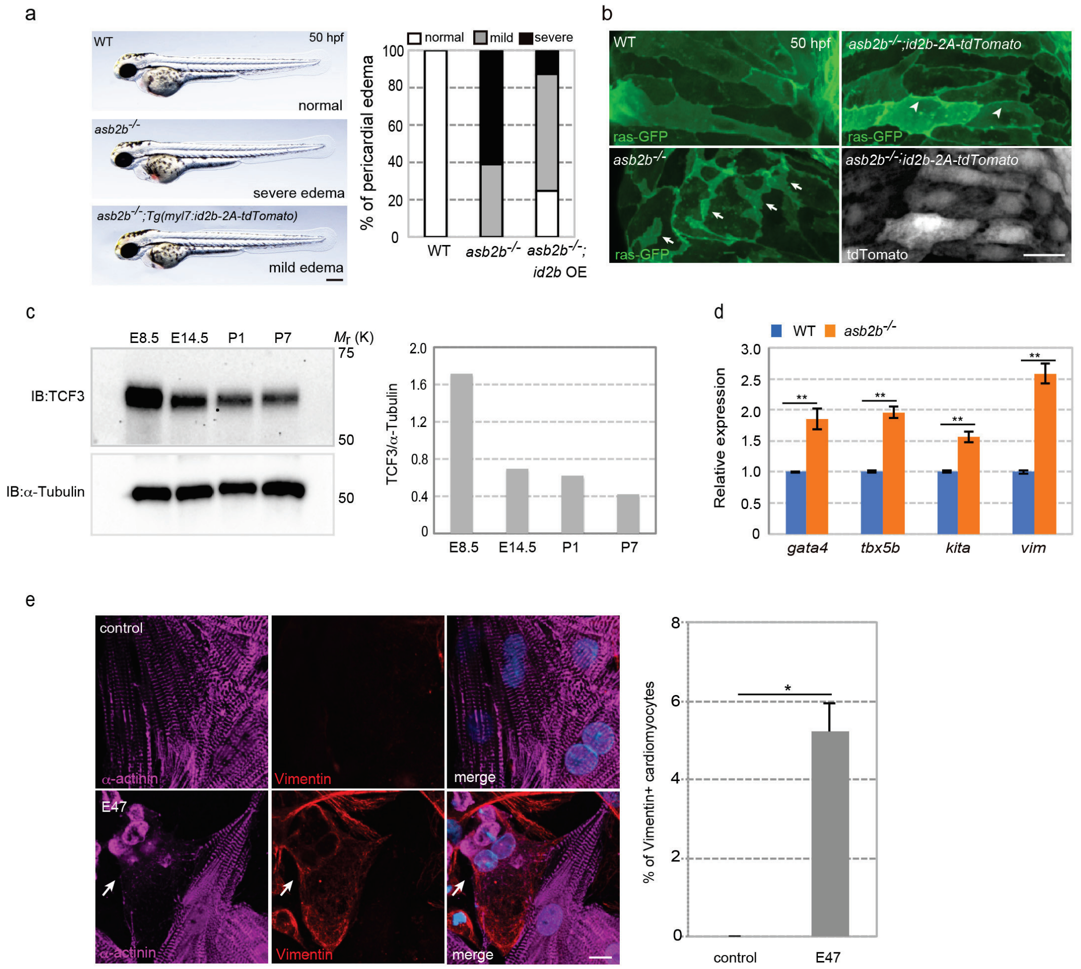

Supplementary Figure 5 - *asb2b* mutant and TCF3 overexpressing cardiomyocytes exhibit a number of phenotypes associated with cardiomyocyte dedifferentiation. (a) Brightfield micrographs of 50 hpf WT, *asb2b* mutant or *Tg(myl7:id2b-p2a-tdTomato) asb2b* mutant embryos in lateral views. Anterior to the left. Scale bar, 200 μm. Number of fish exhibiting pericardial edema (*n*=20 fish). (b) 3D images of 50 hpf *Tg(myl7:ras-GFP)* WT and *asb2b* mutant atria as well as *Tg(myl7:ras-GFP);Tg(myl7:id2b-2A-tdTomato) asb2b* mutant atria. *asb2b* mutant cardiomyocytes exhibit irregular cell-cell borders and membrane protrusions (arrows), whereas *id2b*-overexpressing *asb2b* mutant cardiomyocytes exhibit less severe morphological phenotypes (arrowheads). (c) E8.5, E14.5, P1 and P7 mouse hearts were isolated and TCF3 protein levels were analyzed; relative expression levels shown on the right. (d) Relative mRNA expression (qPCR) of *gata4, tbx5b, kita* and *vim* in isolated 50 hpf WT (blue bar) and *asb2b* mutant (orange bar) hearts (*n*=2 technical replicates, RNA samples were obtained from 500 isolated hearts). (e) Rat NCMs transfected with control or *E47* adenovirus vectors, co-stained for α-actinin (magenta), Vimentin (red) and DAPI (blue). Arrow points to a Vimentin-positive cardiomyocyte. Number of Vimentin-positive Rat NCMs (*n*=2, with averages taken from 50 cardiomyocytes). $^{*}P<0.05$, $^{**}P<0.01$ by one-way analysis of variance (ANOVA) followed by Tukey's HSD test. Error bars, SEM. Scale bars, 20 μm.

Uncropped images related to Fig. 4b

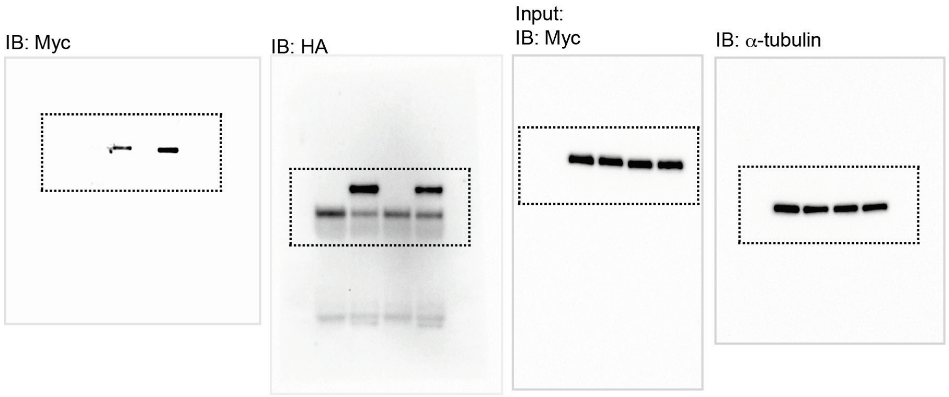

Uncropped images related to Fig. 4c

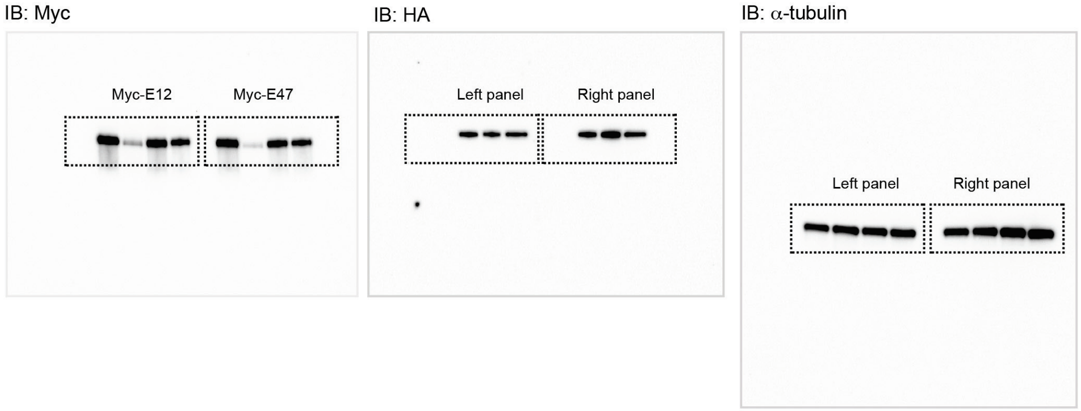

Uncropped images related to Supplementary Fig. 5c

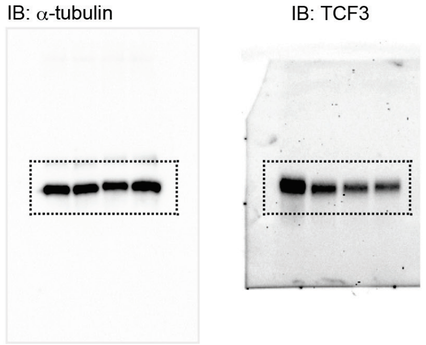

Supplementary Figure 6 – Uncropped images related to western blotting data

Supplementary Tables

Cardiac differentiation marker

ID	Gene Symbol	Gene Name	ratio (mutant/WT)
ENSDART00000102766	*tnnt2a*	*troponin T type 2a*	1.20
ENSDART00000150079	*tnni1b*	*troponin I type 1b*	1.22
ENSDART00000138911	*actn2b*	*actinin, alpha 2b*	1.30
ENSDART00000017677	*ttna*	*titin, tandem duplicate 2*	1.87
ENSDART00000125953	*ttnb*	*titin, tandem duplicate 1*	1.36

Supplementary Fig. 3d - Junctional components

ID	Gene Symbol	Gene Name	ratio (mutant/WT)
ENSDART00000024627	*cdh2*	*cadherin 2*	1.33
ENSDART00000047272	*cx43*	*connexin 43*	0.99
ENSDART00000149871	*cx40.8*	*connexin 40.8*	1.06
ENSDART00000123532	*pkp1a*	*plakophilin 1a*	1.06
ENSDART00000035899	*pkp2*	*plakophilin 2*	0.92
ENSDART00000139110	*pkp3a*	*plakophilin 3a*	1.02
ENSDART00000155685	*pkp4*	*plakophilin 4*	1.18
ENSDART00000019505	*pleca*	*plectin a*	1.22
ENSDART00000144558	*plecb*	*plectin b*	1.16
ENSDART00000108721	*tjp1a*	*tight junction protein 1a*	0.90

Supplementary Fig. 3g - actin regulators

ID	Gene Symbol	Gene Name	ratio (mutant/WT)
ENSDART00000146147	*pak1*	*p21 protein (Cdc42/Rac)-activated kinase 1*	2.55
ENSDART00000090528	*rhoca*	*ras homolog family member Ca*	1.52
ENSDART00000045086	*prkceb*	*protein kinase C, epsilon b*	1.96
ENSDART00000105608	*prkcea*	*protein kinase C, epsilon a*	1.83

TCF3 targets

ID	GeneSymbol	Gene Name	ratio (mutant/WT)
ENSDART00000138887	*cyp11a2*	*cytochrome P450, family 11, subfamily A, polypeptide 2*	3.46
ENSDART00000138702	*MEF2Cb*	*myocyte enhancer factor 2cb*	2.62
ENSDART00000125012	*casp6l1*	*caspase 6, apoptosis-related cysteine peptidase, like 1*	2.60
ENSDART00000074181.2	*zp3.2*	*zona pellucida glycoprotein 3, tandem duplicate 2*	2.09
ENSDART00000108974	*kita*	*kit receptor a*	2.06
ENSDART00000149161	*abcc8*	*ATP-binding cassette, sub-family C (CFTR/MRP), member 8*	1.93
ENSDART00000055537	*socs1a*	*suppressor of cytokine signaling 1a*	1.84
ENSDART00000063775	*mal*	*mal, T-cell differentiation protein*	1.66
ENSDART00000140980	*sema3bl*	*sema domain, immunoglobulin domain (Ig), short basic domain, secreted, (semaphorin) 3bl*	1.62
ENSDART00000153127	*socs3b*	*suppressor of cytokine signaling 3b*	1.56
ENSDART00000137090	*id2a*	*inhibitor of DNA binding 2, dominant negative helix-loop-helix protein, a*	1.40

Supplementary Table 1 – Microarray data. 50 hpf WT and *asb2b* mutant hearts were isolated and RNA expression levels were analyzed by microarray.

Supplementary Fig. 1h

Sample Name	Assay Name	Ct Mean
17.5 hpf WT	*cmlc2*	26.76
17.5 hpf *asb2b* mutant	*cmlc2*	26.01
24 hpf WT	*cmlc2*	23.77
24 hpf *asb2b* mutant	*cmlc2*	23.64
17.5 hpf WT	*vmhc*	23.69
17.5 hpf *asb2b* mutant	*vmhc*	24.09
24 hpf WT	*vmhc*	22.19
24 hpf *asb2b* mutant	*vmhc*	22.10
17.5 hpf WT	*amhc*	29.45
17.5 hpf *asb2b* mutant	*amhc*	29.35
24 hpf WT	*amhc*	26.30
24 hpf *asb2b* mutant	*amhc*	26.84
17.5 hpf WT	*ef1*α	14.97
17.5 hpf *asb2b* mutant	*ef1*α	15.43
24 hpf WT	*ef1*α	15.19
24 hpf *asb2b* mutant	*ef1*α	15.30

Fig. 4a

Sample Name	Assay Name	Ct Mean
50 hpf WT hearts	*socs3b*	25.68
50 hpf *asb2b* mutant hearts	*socs3b*	24.29
50 hpf WT hearts	*id2a*	22.48
50 hpf *asb2b* mutant hearts	*id2a*	22.12
50 hpf WT hearts	*mef2cb*	28.83
50 hpf *asb2b* mutant hearts	*mef2cb*	27.55
50 hpf WT hearts	*ef1*β	23.79
50 hpf *asb2b* mutant hearts	*ef1*β	23.54

Supplementary Fig. 5d

Sample Name	Assay Name	Ct Mean
50 hpf WT hearts	*gata4*	21.08
50 hpf *asb2b* mutant hearts	*gata4*	20.51
50 hpf WT hearts	*tbx5b*	24.14
50 hpf *asb2b* mutant hearts	*tbx5b*	23.49
50 hpf WT hearts	*kita*	25.31
50 hpf *asb2b* mutant hearts	*kita*	24.98
50 hpf WT hearts	*vim*	24.83
50 hpf *asb2b* mutant hearts	*vim*	23.77
50 hpf WT hearts	*ef1*β	18.75
50 hpf *asb2b* mutant hearts	*ef1*β	19.07

Supplementary Table 2 – qPCR data. RNA expression levels were analyzed by RT-qPCR
